# Binding of HSV-1 Glycoprotein K (gK) to Signal Peptide Peptidase (SPP) Is Required for Virus Infectivity

**DOI:** 10.1371/journal.pone.0085360

**Published:** 2014-01-20

**Authors:** Sariah J. Allen, Kevin R. Mott, Yoshiharu Matsuura, Kohji Moriishi, Konstantin G. Kousoulas, Homayon Ghiasi

**Affiliations:** 1 Center for Neurobiology and Vaccine Development, Ophthalmology Research, Department of Surgery, Los Angeles, California, United States of America; 2 Department of Molecular Virology, Research Institute for Microbial Diseases, Osaka University, Osaka, Japan; 3 Department of Microbiology, Faculty of Medicine Yamanashi University, Yamanashi, Japan; 4 Division of Biotechnology and Molecular Medicine, School of Veterinary Medicine, Louisiana State University, Baton Rouge, Louisiana, United States of America; University of Illinois at Chicago, United States of America

## Abstract

Glycoprotein K (gK) is a virion envelope protein of herpes simplex virus types 1 (HSV-1) and 2 (HSV-2), which plays important roles in virion entry, morphogenesis and egress. Two-hybrid and pull-down assays were utilized to demonstrate that gK and no other HSV-1 genes specifically binds to signal peptide peptidase (SPP), also known as minor histocompatibility antigen H13. SPP dominant negative mutants, shRNA against SPP significantly reduced HSV-1 replication *in vitro*. SPP also affected lysosomes and ER responses to HSV-1 infection. Thus, in this study we have shown for the first time that gK, despite its role in fusion and egress, is also involved in binding the cytoplasmic protein SPP. These results also suggest that SPP plays an important role in viral replication and possibly virus pathogenesis. This makes SPP unique in that its function appears to be required by the virus as no other protein can compensate its loss in terms of viral replication.

## Introduction

Signal peptide peptidase (SPP), also known as minor histocompatibility antigen H13, is an aspartyl protease member of the intramembrane cleaving proteases family (I-CLiP), which specializes in the cleavage of signal peptides after their release by signal peptidase (SP) [1], [2]. SPP and SPP-like (SPPL) proteins are evolutionarily conserved in *H. sapiens*, *Rattus norvegicus, Oryza sativa japonica, B. subtilis, Gallus gallus, B. taurus, X. laevis*, *Macaca mulatta, D. rerio*, *D. melanogaster*, *C. elegans*, *S. pombe*, *A. thaliana*, *P. falciparum* [3]–[6] and there exists a 96% amino acid homology between human and mouse SPP [7]. SPP localizes predominantly to the endoplasmic reticulum (ER) and can exist in different forms depending on glycosylation status [8]. Unlike other family members, SPP appears to achieve enzyme activity in the absence of protein cofactors [1], [9], [10]. SPP has been shown to play important roles in extracellular and intracellular signaling events such as cellular surveillance in MHC-I signal peptide processing [2] and has been linked to pathogenic conditions such as Alzheimer's disease [11], certain cancers [12], and human cytomegalovirus, pestivirus, malaria and Hepatitis C infections [13]–[17].

HSV-1 infections are among the most frequent serious viral eye infections in the U.S. and are a major cause of viral-induced blindness [18]–[22]. HSV-1-induced corneal scarring (CS), also broadly referred to as herpes stromal keratitis (HSK), can lead to blindness; thus, HSV-1 is the leading cause of corneal blindness due to an infectious agent in developed countries [21], [23], [24]. In addition to necrotizing HSK, ocular infection with HSV-1 can cause eye disease ranging in severity from blepharitis, conjunctivitis, and dendritic keratitis, to disciform stromal edema [22], [23], [25]–[28]. In the U.S. approximately 500,000 people suffer recurrent ocular HSV episodes annually, requiring doctor visits, medication and corneal transplants in severe cases. Although the HSV-1 gene(s) which are involved in eye disease are presently unknown, we have demonstrated previously that immunization of mice with glycoprotein K (gK), but not with any other known HSV-1 glycoprotein, significantly exacerbates CS and facial dermatitis following ocular HSV-1 infection [29]–[32]. This exacerbation of CS occurs independently of both the virus strain used for infection and the strain of mouse studied [31]. gK is encoded by the UL53 open reading frame and is a highly hydrophobic 338-amino-acid protein with a predicted molecular mass of 37-kDa [32]–[34]. Both gK from HSV-1 and HSV-2 are 338 amino acids long with approximately 84% amino acid homology [33], [35], [36]. Genome wide screenings in both HSV-1 [37] and HSV-2 [38] have shown that gK elicited CD8^+^ IFN-γ responses in mice and humans, respectively.

gK is an essential HSV-1 gene [32]–[34], [39] and single amino acid changes within gK cause extensive virus-induced cell fusion [40]–[43]. Furthermore, gK is an important determinant of cytoplasmic virion envelopment, since viruses lacking gK fail to efficiently acquire a cytoplasmic envelope resulting in a drastic defect in virion morphogenesis, egress and spread [44]–[47]. Deletion of gK results in the formation of extremely rare microscopic plaques indicating that gK is required for efficient virus replication [44], [45], [47], [48], a concept that is supported by the observation that gK-deficient virus can only be propagated on complementing cells that express gK [44], [45]. As gK is essential to HSV-1 infectivity, we had previously analyzed its contribution to CS using recombinant viruses (rather than deleting the *gK* gene) with two extra copies of *gK* and found that similar to gK immunization, this recombinant virus caused elevated levels of CS in both mice and rabbits [49]. We have also shown that an elevation of anti-gK antibody in individuals with a history of HSV-1 recurrence is correlated with increased severity of eye disease [50].

In this study we show for the first time that: 1) HSV-1 gK binds to SPP and 2) SPP is required for virus infectivity. Despite the seriousness of ocular herpes infection, no drug has been FDA approved for prevention of ocular recurrences. Thus, blocking SPP activity or binding to viral glycoproteins (such as gK) by targeted therapeutics may represent a clinically effective and expedient approach to the reduction of viral replication and the resulting pathology.

## Materials and Methods

### Cells and viruses

Vero and HeLa cells were obtained from American type culture collection (ATCC). RS (rabbit skin) cells (from Steven L Wechsler) was described previously [51]. HeLa and Vero cells were grown in DMEM media plus 10% fetal bovine serum (FBS), while RS cells were grown in MEM media plus 5% FBS, while. Triple plaque-purified HSV-1 strain McKrae was grown in RS cell monolayers as described previously [32]. V5-tagged gK recombinant viruses in KOS background (gKV5DI, gKV5DII, gKV5DIII, and gKV5DIV) were grown as described previously [52].

### Two hybrid system

We performed a bacterial two-hybrid using the BacterioMatch Two-Hybrid System (Stratagene, La Jolla, CA) and a mouse brain plasmid cDNA library (Stratagene). The bait plasmid pBT expressing a λ repressor (λcI)-fused gK protein and the target plasmid pTRG expressing the α-subunit of RNA polymerase fused to cDNA library-encoded proteins were used in the study. We used an *E.coli* reporter strain containing the two reporter genes LacZ and Carbenecillin-resistance (Carb^r^) under the control of the λcI/α-subunit of RNA polymerase. Additionally, the pBT plasmid, the pTRG plasmid and the *E.coli* reporter strain contained the chloroamphenicol (Cam^r^), tetracycline (Tet^r^) and kanamycin (Kan^r^) resistance genes, respectively. To construct the pBT-gK, a cDNA encoding gK was amplified by polymerase chain reaction (PCR) using specific primers containing EcoRI/XhoI sites and inserted into the corresponding sites in the pBT bait plasmid. The mouse brain cDNA library was amplified, harvested and final plasmid DNA (pTRG-cDNA mouse brain library) purification conducted according to manufacturer's protocol. The *E.coli* reporter strain was transformed with pBT-gK and cDNA library cloned into pTRG and transformants were selected on Carb + Cam + Tet + Kan supplemented LB-Agar plates. The putative positive colonies were further tested for Lac Z activity by replica plating these clones onto X-gal indicator plates (Cam + Tet + Kan +X-gal + β-galactosidase inhibitor LB-Agar) followed by screening for the blue color indicative of Lac Z expression. The mouse brain library plasmids were recovered from the positive colonies and the inserted target cDNA was sequenced using pTRG plasmid-specific primers as described in the manufacture's protocols. NCBI-BLAST analysis [53] was performed on collected sequences and putative genes identified (Figure S1).

### Construction and expression of c-myc-gK and HA-SPP

The gK and SPP constructs used in this study are shown in Figures S2 and S3, respectively. In Figure S2, a schematic diagram of full-length gK with an in-frame c-myc tag at the carboxy terminus is shown. Figure S3 shows a schematic diagram of full-length SPP with an in-frame HA tag and ER retention signal also located at the carboxy terminus as we described previously [16]. gK with c-myc tag was synthesized (GenScript, Piscataway, NJ) and inserted into BamHI site of pcDNA3.1 and sequences were verified with standard dideoxy sequencing at the UCLA Genotyping and Sequencing core. Amaxa nucleofactor kit R (Lonza, Allendale, NJ) was used to transfect 10^6^ HeLa or Vero cells with plasmid DNA cocktail containing both HA-SPP and c-myc-gK in a ratio of 1∶1 in accordance with manufacturer instruction. Protein expression was monitored over 5 days using Coomassie blue protein staining and Western blotting. Antibodies against HA and c-myc (GenScript), were diluted according to manufacturer instruction in the total Western HRP kit (GenScript). Optimum c-myc-gK and HA-SPP expression and recovery was determined to be 48–72 hr post-transfection.

### Construction and expression of SPP shRNA constructs

shRNAs against SPP were created using the Knockout single vector inducible RNAi system (Clontech, Mountain View, CA). Briefly, SPP siRNA oligonucleotides were designed using siRNAdesigner (www.clontech.com). The shRNAs chosen correspond to SPP nucleotide locations 409–430 (#5/6); 644–666 (#11/12); 1134–1157 (#19/20) and a scramble of #11/12. The four shRNA were synthesized and ligated into pSingle-tTS-shRNA (Invitrogen) via XhoI and MluI restriction sites and the sequence was verified using standard dideoxy sequencing. RS cells were grown to 70% confluency on Lab-Tex chamber slides (BD Falcon, San Jose, CA) and transfected with either SPP shRNA or scramble shRNA using Lipofectamine-2000 (Invitrogen, Carlsbad, CA) for 8 hr followed by addition of plasmid inducer doxycycline for 12 hr prior to HSV-1 infection according to manufacturer instruction. Cells were infected with 0.1 PFU of HSV-1 strain McKrae for 1 hr at 37°C, virus was then removed with three 1X PBS washes and normal growth media + shRNA inducer replaced for 2, 4, 6, 8, 20 or 40 hr post-infection (PI). At each time point virus titer was measured via standard plaque assay on RS cells as we described previously [31]. Briefly, 100 μL aliquots of 10-fold serial dilutions were placed on confluent monolayers of RS cells in 24-well plates, incubated at 37°C for 1 hr and overlaid with medium containing 1% methylcellulose. The plates were incubated at 37°C for 3 days and stained with 1% crystal violet, and the viral plaques were counted.

### Construction and expression of SPP dominant negative mutants

We previously constructed two mutant forms of SPP in which enzymatically critical Asparagine residues were mutated to Alanine at positions 219 (D219A) and 265 (D265A) [16]. These dominant negatives are also HA tagged with ER-retention signals at the carboxy terminus in pcDNA3.1 vector as shown in Figure S3 and as we described previously [16]. RS cells were grown to confluency in Lab-Tex chamber slides and transfected with SPP-HA, D219A-HA or D265A-HA plasmids using Lipofectamine-2000 (Invitrogen). Transfection was allowed to proceed for 24 hr followed by infection with 0.1 PFU of HSV-1 strain McKrae as described above. Cells were grown for 12, 24 or 48 hr PI and HSV-1 titer measured by standard plaque assay on RS cells as described above.

### Immuoprecipitation (IP)

HeLa or Vero cells were transfected with c-myc-gK and HA-SPP as described above and were harvested at 48 hr post-transfection. Cells were lysed with lysis buffer included in the Classic IP Kit (Pierce, Rockford, IL) and 600 μg cellular extract was incubated with Dynabeads-G (Invitrogen) which were pre-bound to either HA, c-myc, irrelevant His-antibody (Invitrogen) or SPP (Abcam) antibody. Incubation proceeded for 1 hr at RT and beads were washed 5X with lysis buffer followed by kit-supplied elution buffer and finally SDS-PAGE analysis and Western blotting using the reverse antibodies that was used for IP.

### Colocalization and virus detection by immunocytochemistry (ICC)

HeLa, Vero and RS cells were grown to confluency on Lab-tek chamber slides and infected with gK-V5-DII recombinant HSV-1 for 24 hr as we described previously [52]. Infected cells were fixed with 4% paraformaldehyde for 1 hr at 4°C followed by 20 minutes incubation in serum free protein block (Dako, Carpentaria, CA) at room temperature. Rabbit anti-SPP (Abcam) was diluted according to manufacturer instructions and incubated on slides overnight at 4°C. Slides were then washed and incubated with anti-V5-FITC, anti-FITC Alexa Fluor 488 and anti-rabbit Alexa Fluor 594 (Invitrogen) for 1 hr at RT. Washed slides were air dried and mounted with 4′,6-diamidino-2-phenylindole (DAPI) prolong Gold (Invitrogen). The fluorophores were imaged in separate channels with a Zeiss ApoTome-equipped Axio Imager Z1 (Carl Zeiss Microimaging). For anti-HSV-1-gC-FITC staining, RS or Vero cells were grown to confluency on chamber slides and transfected with shRNA construct or dominative negative construct as described above, followed by infection with 0.1 PFU/cell of McKrae for 24 hr. Fixative, blocking and mounting was the same as above except with anti-HSV-1-gC-FITC antibody incubation at a 1∶100 dilution overnight at 4°C (Genway, San Diego, CA). For organelle straining, RS cells were grown to confluency on chamber slides and transfected with shRNA as described above followed by infection with 0.1 PFU/cell of McKrae for 24 hr. Fixative, blocking and mounting was the same as above except with rabbit polyclonal antibodies (Abcam) against LAMP (ab24170), EEA1 (ab2900) or Calnexin (ab22595) diluted according to manufacturer instruction followed by anti-rabbit Alexa Fluor 594 secondary antibody at 1∶200 dilution.

### Fluorescent-activated cell sorting (FACS)

RS cells were transfected with SPP shRNA 11/12 as above followed by infection with 0.1 PFU/cell of HSV-1 strain McKrae for 24 hr or mock-infected. Infected or mock-infected cells were harvested via centrifugation and stained with annexin-V PE mAb (eBioscience, San Diego, CA). Stained cells were washed 2X with FACS buffer (1X PBS with 0.1% sodium azide), resuspended in 4% paraformaldehyde, and analyzed using a multicolor five-laser LSR II instrument (Applied Biosystems, Foster City, CA).

### Gene expression analyses

qRT-PCR was performed as follows: at various times post infection, total RNA was extracted, and 1,000 ng of total RNA was reverse transcribed as we have described previously [54]. The differences in the expression levels of mRNAs were evaluated using custom-made TaqMan gene expression primers against ICP0, tK, gB and gK with optimized primer and probe concentrations (Applied Biosystems). Primer probe sets consisted of two unlabeled PCR primers and the FAM^TM^ dye-labeled TaqMan MGB probe formulated into a single mixture. Additionally, all cellular amplicons included an intron-exon junction to eliminate signal from genomic DNA contamination. The assays used in this study were as follows: 1) gB specific primers (forward, 5′-AACGCGACGCACATCAAG-3′; Reverse - 5′-CTGGTACGCGATCAGAAAGC-3′; and Probe - 5′-FAM-CAGCCGCAGTACTACC-3′). Amplicon Length  = 72 bp; 2) ICP0 specific primers (forward, 5′- CGGACACGGAACTGTTCGA-3′; reverse, 5′-CGCCCCCGCAACTG-3′; and probe, 5′-FAM-CCCCATCCACGCCCTG-3′). Amplicon length  = 111 bp; 3) TK specific primers (forward, 5′- CAGTAGCGTGGGCATTTTCTG-3′; reverse primer, 5′-CCTCGCCGGCAACAAAA-3′; and probe, 5′-FAM-CTCCAGGCGGACTTC-3′). Amplicon length  = 59 bp; and 4) gK specific primers (forward, 5′-GGCCACCTACCTCTTGAACTAC-3′; reverse primer, 5′-CAGGCGGGTAATTTTCGTGTAG-3′; and probe, 5′-FAM-CAGGCCGCATCGTATC-3′). Amplicon length  = 82 bp. As an internal control, a set of GAPDH primers from Applied Biosystems (ASSAY I.D. m999999.15_G1 – Amplicon Length  = 107 bp) was used.

In some experiments the relative copy numbers for ICP-0, gB, and gK expressions were calculated using standard curves generated from the plasmids pGem-ICP0, pAc-gB1, and pAC-gK1, while in other experiments of ICP-0, tk, gB, and gK expressions were normalized to the levels present 1 hr after virus is first added to the cell monolayer (the adsorption period), a time is routinely taken as t = 0. In all experiments, GAPDH was used for normalization of transcripts. qRT-PCR was performed using an ABI ViiA7 sequence detection system (Applied Biosystems) in 384-well plates. The threshold cycle (CT) values, which represent the PCR cycles at which there is a noticeable increase in the reporter fluorescence above baseline, were determined using ViiA7 RUO software.

## Results

### HSV-1 gK binds to SPP

We hypothesized that gK might exert its previously demonstrated pathogenic functions via interactions with one or more cellular proteins. Briefly, we performed a two-hybrid screening assay using the BacterioMatch Two-Hybrid System (Stratagene, San Diego, CA). In this assay, gK was used as a bait to probe a mouse brain cDNA library to find cellular proteins that interact with gK. A total of 1×10^6^ independent cDNA clones were screened and upon sequencing we observed a significant homology to all isoforms of SSP (Fig. S1) suggesting a possible interaction of gK and SPP.

**Figure 1:**
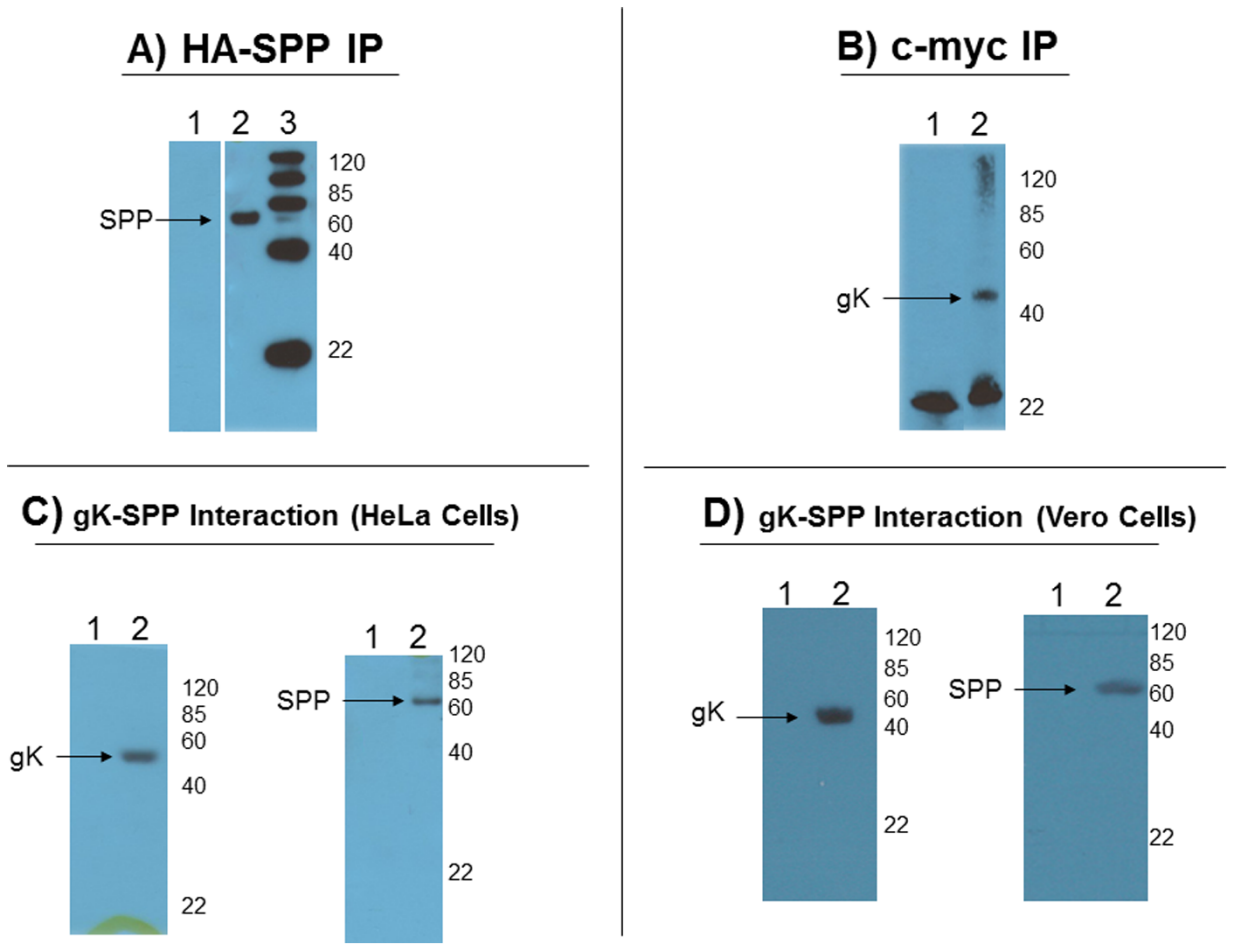
Binding of gK to SPP *in vitro*. HeLa cells were transfected with c-myc-gK and HA-SPP plasmids at a 1∶1 ratio for 48 hr. A) Expression and pull-down of HA-SPP. Cellular lysates were incubated with anti-HA antibody bound to IgG beads and the resulting IP was subjected to Western blot analysis with anti-HA antibody. Lane 1 shows untransfected HeLa cells and no HA-SPP band, while Lane 2 shows HA-SPP correctly immunoprecipitating from transfected lysates. Lane 3 is protein size marker; B) Expression and pull-down of c-myc-gK. Cellular lysates were incubated with anti-c-myc antibody bound to IgG beads and the resulting IP was subjected to Western blot analysis with anti-c-myc antibody. Lane 1 shows untransfected HeLa cells no c-myc-gK band, while Lane 2 shows c-myc-gK correctly immunoprecipitating from transfected lysates. Protein sizes are indicated; and C/D) Co-immunoprecipitation of gK and SPP; C) HeLa Cells. Left panel: Cellular lysates were incubated with anti-HA antibody bound to IgG beads and the resulting IP was subjected to Western blot analysis with anti-c-myc antibody. Lane 1 shows untransfected HeLa cells and no gK band, while Lane 2 shows a successful pull-down of gK by anti-HA antibody. Right panel: Cellular lysates were incubated with anti-c-myc antibody bound to IgG beads and the resulting IP was subjected to Western blot analysis with anti-HA antibody. Lane 1 shows untransfected HeLa cells and no SPP band, while Lane 2 shows a successful pull-down of SPP by c-myc-gK. Protein sizes are indicated; D) Vero Cells. Left panel: Cellular lysates were incubated with anti-HA antibody bound to IgG beads and the resulting IP was subjected to Western blot analysis with anti-c-myc antibody. Lane 1 shows untransfected Vero cells and no gK band, while Lane 2 shows a successful pull-down of gK by anti-HA antibody. Right panel: Cellular lysates were incubated with anti-c-myc antibody bound to IgG beads and the resulting IP was subjected to Western blot analysis with anti-HA antibody. Lane 1 shows untransfected Vero cells and no SPP band, while Lane 2 shows a successful pull-down of SPP by c-myc-gK; E) HSV-1 infected lysates subjected to IP with total HSV-1 serum followed by Western blot with total HSV-1 serum pulled down many proteins; F) HSV-1 Infected lysates subjected to IP with total HSV-1 serum followed by Western blot with anti- SPP antibody did not pull down SPP; and G) HSV-1 infected lysates subjected to IP with anti-SPP antibody followed by Western blot with total HSV-1 serum did not pull down any HSV-1 reacting proteins. Protein sizes are indicated.

To confirm the bacterial two-hybrid result of gK binding to SPP, we first used a pull-down approach. Cellular extracts from HeLa cells transiently expressing HA-SPP, c-myc-gK or both plasmids were immunoprecipitated using protein G beads bound to either anti-myc, anti-HA, or the irrelevant anti-His antibody. Immunoprecipitates were subjected to Western analysis to detect c-myc-gK (using c-myc Ab) or HA-SPP (using HA Ab). We created the tagged myc-gK plasmid as there is no commercially available antibody against gK. Furthermore, we have previously failed in attempts to make antibody against either full-length gK or gK peptide fragments in multiple hosts (mouse, rabbit and chicken). Figure 1A demonstrates pull-down of HA-SPP by anti-HA antibody, while Figure 1B demonstrates pull-down of c-myc-gK by anti-c-myc antibody demonstrating that both proteins can be individually immunoprecipitated using this system. Figures 1C and 1D demonstrate successful co-immunoprecipitation of HA-SPP and c-myc-gK in two cell lines. Figure 1C shows gK-SPP interaction via both pull-down of c-myc-gK by anti-HA immunoprecipitation and pull-down of HA-SPP by anti-c-myc immunoprecipitation in HeLa cells. Neither HA-SPP nor c-myc-gK were pulled down in untransfected HeLa cells (lane 1 in each figure), or by irrelevant His-antibody (data not shown). Our HeLa cell co-immunoprecipitation results were also confirmed in Vero cells (Fig. 1D). These experiments confirm our two-hybrid analysis and further suggest that gK can bind to SPP *in vitro*.

It is possible that SPP could bind to other HSV-1 proteins and thus our result would not be an interaction specific to gK. To address this possibility we performed an additional IP with RS cells that had been infected with 0.1 and 1.0 PFU/cell of HSV-1 strain McKrae to probe for interaction of other viral proteins with SPP. At 24 hr PI, infected cells were subjected to IP using SPP antibody or total anti-HSV-1 antibody with mock serum as a control. This anti-HSV-1 antibody recognizes many HSV-1 genes including all major HSV-1 glycoproteins (i.e., gB, gC, gD) but not gK. The results demonstrate that the total anti-HSV-1 antibody was able to pull down many HSV-1 proteins, but not SPP (not shown). In addition, the IP against SPP was not able to pull down any HSV-1 reacting proteins (not shown). Taken together these data demonstrate that gK is the only HSV-1 gene that binds to SPP *in vitro.*

### Virus-expressed gK colocalizes with cellular SPP *in vitro*

**Figure 2:**
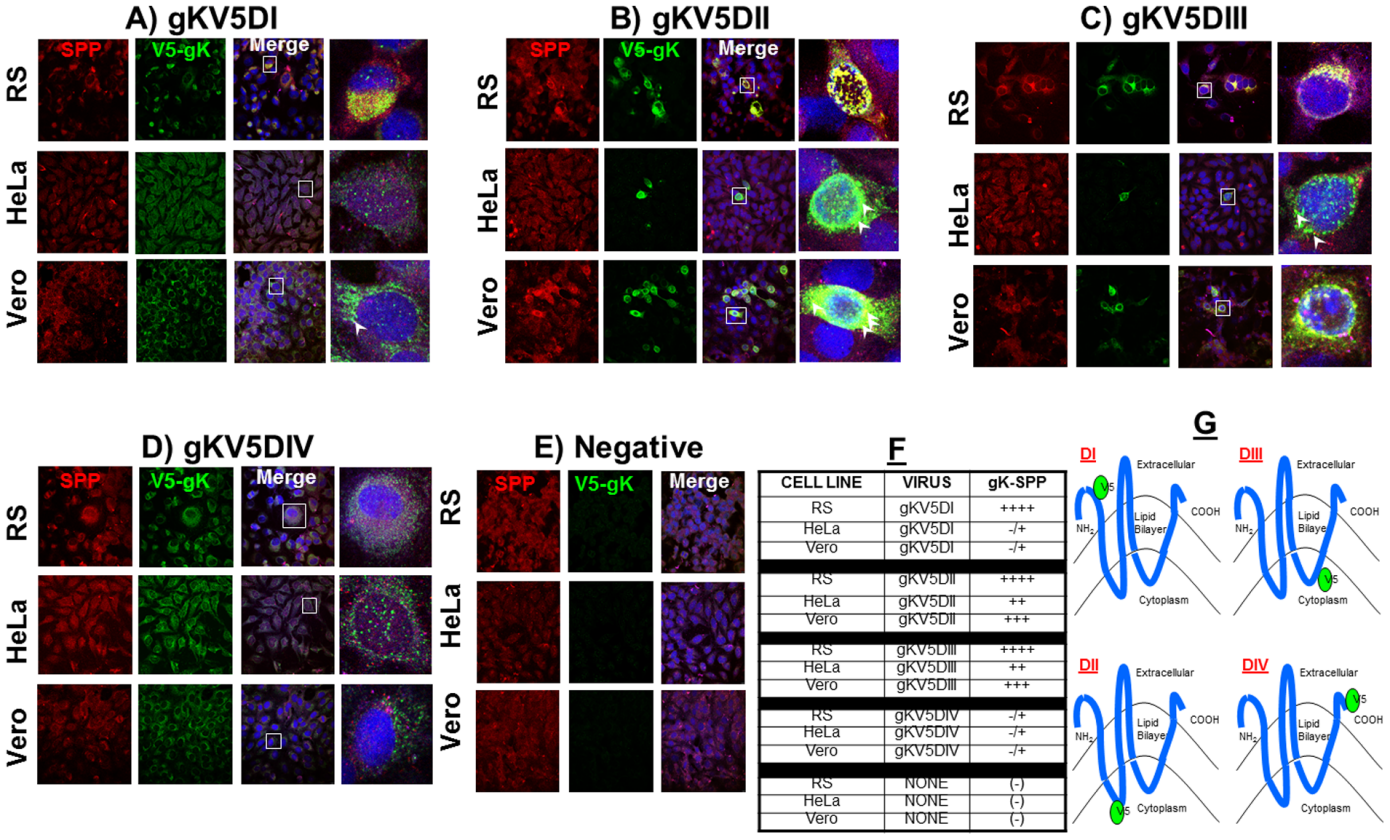
gK colocalizes with SPP *in vitro.* HeLa, Vero and RS cells were infected with 100 PFU/cell of each of four different recombinant HSV-1 expressing V5 tagged gK. Infection was allowed to proceed for 24 hr and slides were fixed, blocked and stained with mouse-anti-V5-FITC (green), rabbit-anti-SPP-TRITC (red) and DAPI nuclear stain (blue). Photomicrographs are shown at 40X direct magnification and colocalization was visualized as yellow. Panels: A) HeLa, Vero and RS cells were infected with gKV5DI; B) HeLa, Vero and RS cells were infected with gKV5DII; C) HeLa, Vero and RS cells were infected with gKV5DIII; D) HeLa, Vero and RS cells were infected with gKV5DIV; E) Mock-infected HeLa, Vero and RS cells; F) Qualitative assessment of colocalization of V5-gK and SPP in all cell lines; and G) V5-gK constructs showing the domain location of the V5 tag within the gK protein. Arrows point to less obvious areas of colocalization. In each panel the top cell line is RS cells, the middle panel is HeLa cells and the bottom panel is Vero cells.

To explore if gK and cellular SPP co-localize within the HSV-1 infected cells, HeLa, Vero and RS cells were infected with four different HSV-1 recombinant viruses expressing V5 in-frame in each of the four proposed domains of gK (Figure 2) [52]. These recombinant viruses differ in the placement of the V5 tag; DI and DIV have V5 on the luminal side while DII and DIII have the tag on the cytoplasmic side (Figure 2G). Cells were infected with each virus individually and IHC was performed using antibodies against SPP and V5 as described in Materials and Methods. We detected strong colocalization between V5-gK and endogenous SPP with DI (Figure 2A), DII (Figure 2B) and DIII (Figure 2C) infected RS cells. In contrast, HeLa and Vero cells had the strongest colocalization with only DII and DIII viruses (Figures 2B and 2C), while we observed minor colocalization with the DIV virus in all cells lines tested (Figure 2D) and no colocalization in uninfected control cells (Figure 2E). The gK-SPP interaction in all cell lines infected with the 4 recombinant viruses are quantified and shown in Figure 2F. These results validate our immunoprecipitation results, since cellular SPP and HSV-1 expressed gK colocalize within the cell.

### SPP shRNA reduces HSV-1 replication *in vitro*

**Figure 3:**
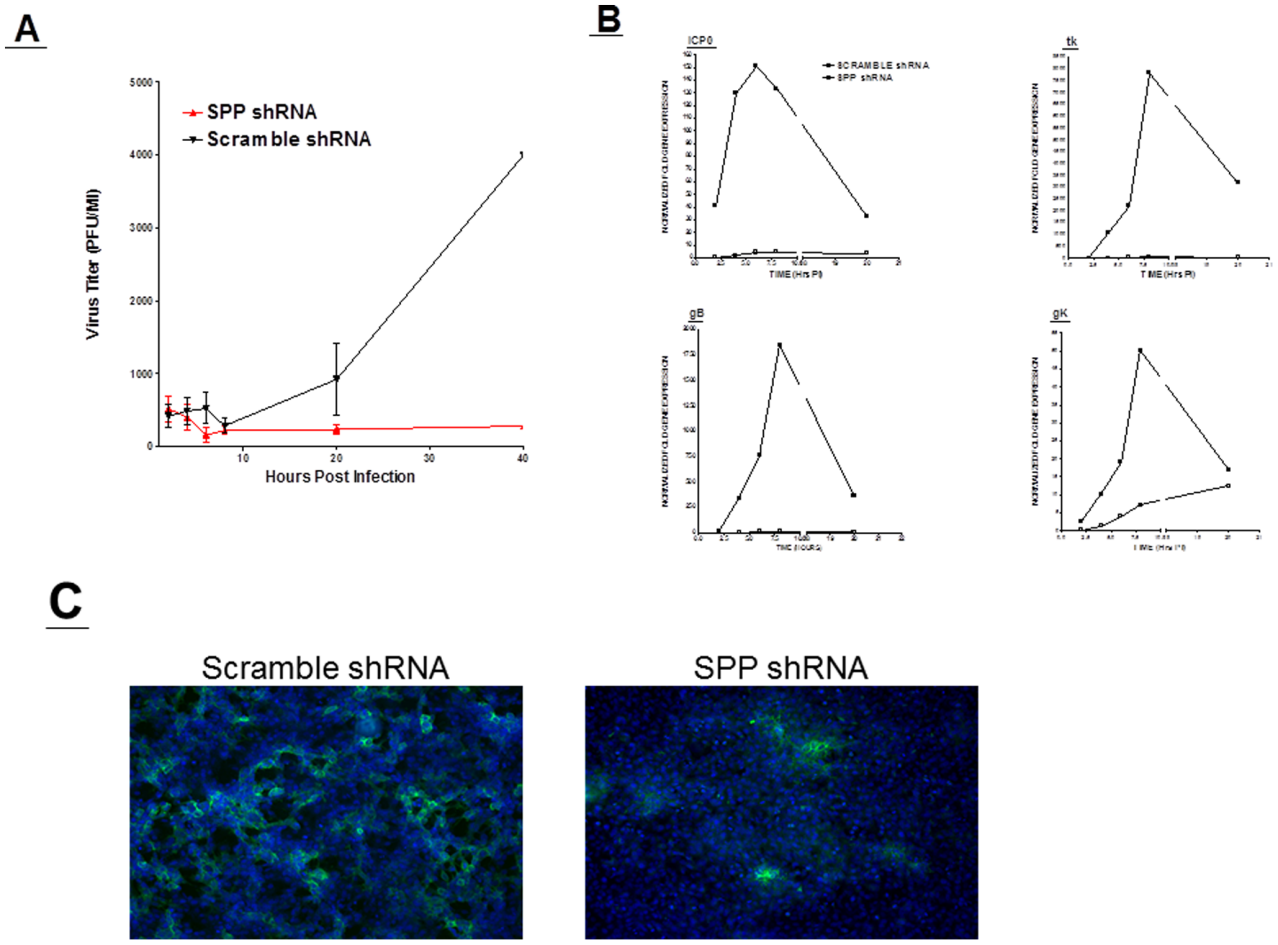
Blocking HSV-1 replication *in vitro* by SPP shRNA. A) Viral Titer is reduced by SPP knockdown. RS cells were transfected for 24 hr with either SPP shRNA or scramble shRNA and infected with 0.1 PFU/cell of HSV-1 strain McKrae. Titers were measured by standard plaque assays at 2.5, 5, 7.5, 10, 20 and 40 hr PI. Each point represents the mean ± SEM from 3 independent experiments per time point; B) HSV-1 gene expression is reduced by SPP knockdown. RS cells were transfected and infected as above. Transfected and infected cells were harvested 2, 4, 6, 8 and 20 hr PI, RNA extracted and cDNA synthesized. Expression of tk, gB and gK were measured using qRT-PCR and each point represents the mean ± SEM from 3 independent experiments; and C) HSV-1 protein expression is reduced by SPP knockdown. RS cells were transfected and infected as in A for 24 hr PI. Cells were stained with anti-HSV-1-gC-FITC (green) and costained with DAPI (blue). Photomicrographs are shown at 10X magnification.

It has been previously shown that small interfering RNA targeted to SPP reduced the production of infectious HCV particles [55]. To explore the possibility that a reduction in SPP production would effect HSV-1 replication, we constructed three shRNA plasmids against SPP as described in Materials and Methods. In a pilot experiment we tested the efficacy of these shRNA against HSV-1 replication *in vitro,* and determined that shRNA construct 11/12 was the most potent in reducing SPP expression in Vero, RS, and HeLa cell lines (Figure S4). We next tested this shRNA construct in RS cells to determine if SPP knockdown had any effect on HSV-1 viral replication. Cells were transfected with SPP shRNA, followed by infection with 0.1 PFU of HSV-1 strain McKrae and HSV-1 titer was measured by standard plaque assays. After 8 hr PI the SPP shRNA plasmid began to significantly reduce viral replication *in vitro* when compared to scrambled SPP shRNA plasmid (Fig 3A). Thus, these results suggest that SPP is needed for efficient HSV-1 infectivity.

During the course of primary HSV-1 infection, gene expression is synchronized in a cascade fashion. Thus, to determine if the observed reduction in virus replication described above (Fig. 3A) affected different classes of HSV-1 gene expression, we investigated the effect of SPP inhibition on HSV-1 tk, gB, and gK expression at various times PI. RS cells were transfected with shRNA plasmids followed by infection with HSV-1 strain McKrae as described above. qRT-PCR was performed on total RNA isolated from transfected-infected RS cells and real time analysis performed. We detected significant reductions in expression of tk, gB, and gK from 2.5 to 20 hr in cells treated with SPP shRNA compared to cells treated with control scramble shRNA (Fig 3B). These results indicate that tk and gB expression is also impaired when SPP expression is blocked.

To confirm our titration and gene expression studies, we next performed ICC against HSV-1 during treatment with shRNA against SPP. RS cells were transfected and infected as above, and at 24 hr PI subjected to ICC using anti-HSV-1-gC antibody. We observed reduced staining for HSV-1 in SPP shRNA transfected RS cells compared to scramble shRNA control (Fig. 3C). We also observed a much more confluent monolayer in SPP shRNA transfected and infected cells indicating reduced cellular lysis as compared to SPP scramble shRNA transfected and infected cells. To demonstrate that the reduction in viral replication and gene expression was not due to higher apoptosis, we performed qRT-PCR on RS cells transfected with SPP shRNA and infected with HSV-1 and compared it to RS cells infected with HSV-1 alone and mock-treated control cells. We observed a significant reduction in apoptosis in the presence of the SPP shRNA plasmid compared to cells infected with HSV-1 alone (Figure S5). This suggests that shRNA against SPP is not increasing cell death and is actually protective of HSV-1 induced apoptosis. Taken together, our RNA interference studies suggest that SPP is required for efficient HSV-1 infectivity.

**Figure 4:**
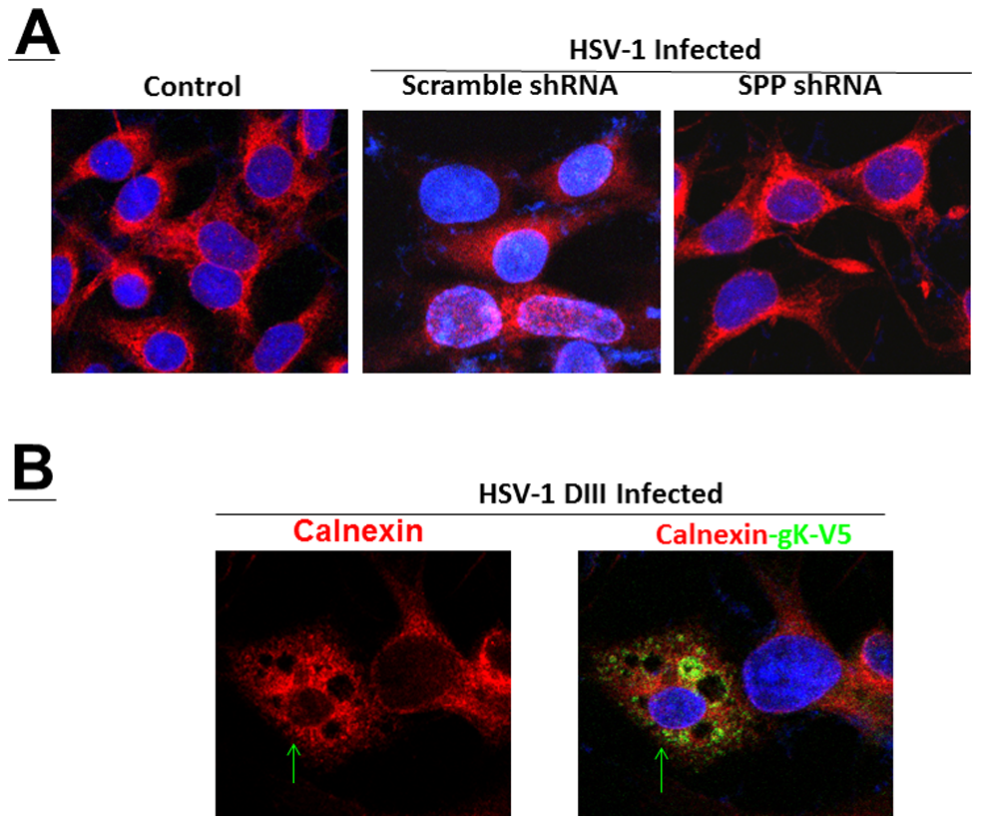
HSV-1 induces ER morphology changes which can be blocked by SPP shRNA. A) RS cells were grown to confluency on chamber slides and transfected with SPP shRNA or scramble shRNA followed by infection with 1 PFU/cell of HSV-1. At 24 hr PI, slides were fixed, blocked and stained with rabbit-anti-calnexin-TRITC (red) and DAPI nuclear stain (blue). Photomicrographs are shown at 40X direct magnification. HSV-1 infection induces condensation of ER while treatment with SPP shRNA restores normal ER morphology. B) RS cells were infected with gKV5DIII and stained for V5 (green) and calnexin (red). Arrow indicates HSV-1 infected cell.

The effect of blocking SPP on intercellular transport properties of the HSV-1 in the ER, lysosomes and endosomes was evaluated in HSV-1 infected RS cells. RS cells were transfected with SPP shRNA or scramble shRNA followed by infection with HSV-1. Transfected-infected cells were monitored by immunofluorescence or immunocytochemistry for the effect of SPP shRNA on morphological properties of the ER, lysosomes and endosomes. We detected significant differences between infected cells in presence of SPP shRNA compared with cells transfected with scramble shRNA and infected which were similar to uninfected cells (Fig. 4A). Loss of SPP function resulted in the loss of discrete punctate structures representing the endosomes around the nuclear rim. RS cells were also infected with V5-tagged gK recombinant virus gKV5DIII. Double staining for V5-gK and ER is shown in Figure 4B, the arrow indicates a HSV-1 infected cell. Our results show that gK also localizes in the ER (yellow), which marks the primary site for a direct interaction between gK and SPP.

**Figure 5:**
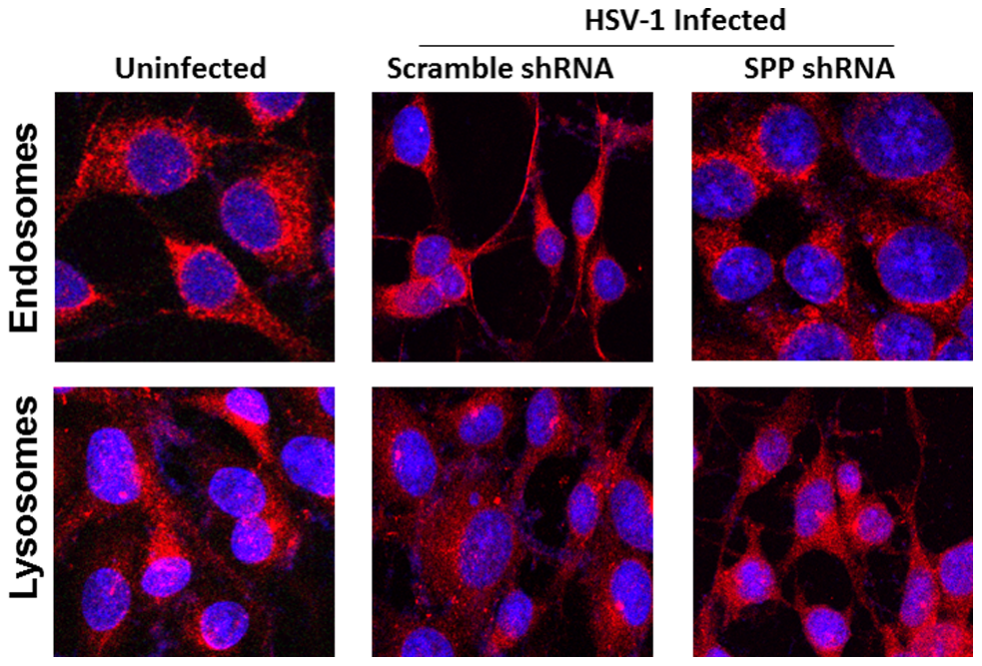
Effect of blockage of gK interaction with SPP on intercellular transport properties of the HSV-1 in the lysosome and endosome. RS cells were grown to confluency on chamber slides and transfected with SPP shRNA or scramble shRNA followed by infection with 1 PFU/cell of HSV-1. At 24 hr PI, slides were fixed, blocked and stained with rabbit-anti-EEA1-TRITC (red) for endosome or rabbit-anti-LAMP-TRITC (red) for lysosome. DAPI was used for nuclear staining (blue). Photomicrographs are shown at 40X direct magnification.

With regards to endosomes we did not detect differences between mock infected control and infected cells treated with SPP shRNA or scramble shRNA (Fig. 5). However, we detected striking difference in the lysosomes between mock infected control and infected cells treated with SPP shRNA compared with cells transfected with scramble shRNA and infected with HSV-1 (Fig. 5). In cells transfected with scramble shRNA and infected, lysosomes were less visible upon infection and mostly located around the nuclear rim and near the ER. Upon shRNA downregulation of SPP the lysosomal population becomes very similar to the uninfected cells. In this latter case the lysosomes were uniformly distributed in the cytoplasm. Thus, our results suggest that SPP regulates lysosomes and ER in response to HSV-1 infection.

### SPP dominant negative mutants reduce HSV-1 replication *in vitro*

**Figure 6:**
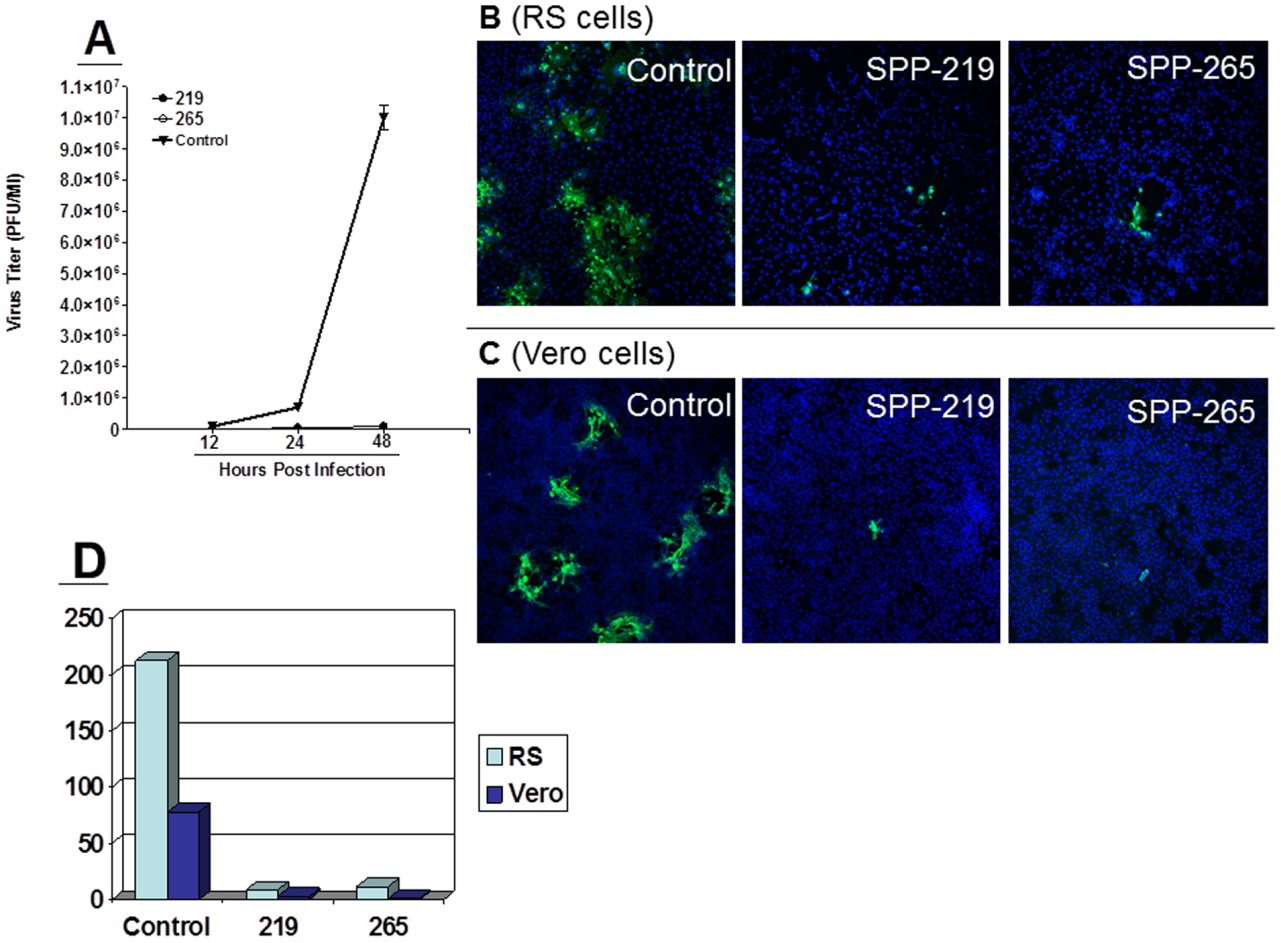
Blocking HSV-1 replication *in vitro* by SPP dominant negative mutants. A) Viral Titer is reduced by SPP dominant negatives. RS cells were transfected for 24 hr with either dominant negative SPP D219A, SPP 265A or wild-type SPP and infected with 0.1 PFU/cell of HSV-1 strain McKrae. Titers were measured by standard plaque assay 12, 24 and 48 hr PI. Each point represents the mean ± SEM from 3 independent experiments per time point; and B/C/D) HSV-1 protein expression is reduced by SPP knockdown. RS cells (B) and Vero cells (C) were transfected and infected as in A. At 24 hr PI, cells were stained with anti-HSV-1-gC-FITC (green) and costained with DAPI (blue). Photomicrographs are shown at 10X magnification. D) Quantification of HSV-1 positive cells from (B) and (C).

Dominant negative mutants are a powerful tool for studying enzyme function as they complex with endogenous proteins, inactivating the wild-type cellular proteins within the same cell. The active site mutants, Asp219 (D219A) and Asp265 (D265A) (constructs shown in Fig. S3), in which the catalytic aspartate residues are mutated to alanine, have been shown to be dominant negative inhibitors of endogenous SPP activity [1], [56]. These catalytic aspartates are highly conserved in all aspartic proteases and their mutation destroys proper coordination of a water molecule in the enzymatic active site, thereby destroying the acid-base reaction and rendering the mutants unable to catalyze their substrate. Importantly, these mutations do not affect substrate binding. To determine if the effect of these dominant negative mutants would confirm our shRNA results, RS cells were transfected with mammalian expression plasmids containing HA-tagged dominant negative SPP plasmids, D219A or D265A, followed by infection with 0.1 PFU of HSV-1 strain McKrae. The kinetics of virus replication were quantitated by determining the amount of infectious virus at various times PI using a standard plaque assay as described in Materials and Methods. Replication of HSV-1 in cells transfected with D219A or D265A was significantly lower than the control group at various times PI (Fig. 6A). In addition, D265A blocked virus replication more efficiently then D219A (Fig. 6A). These results are consistent with our shRNA results (Fig. 3, above) demonstrating that blocking SPP catalytic ability, but not substrate binding, significantly reduces HSV-1 virus replication *in vitro*.

To confirm our titration results with dominant negative mutants, we transfected and infected monolayers of RS and Vero cells as above and subjected them to ICC with anti-HSV-1-gC antibody. Representative photomicrographs of infected RS cells (Fig. 6B, top panels) and Vero cells (Fig. 6C, bottom panel) are shown. In both RS and Vero cells, the presence of D219A and D265A reduced the amount of HSV-1 positive cells compared with control groups (Fig. 6B and C). The positive cells per slide were quantitated and indicate that there is approximately a 10 fold reduction in HSV-1 signal in cells transfected with SPP dominant negative mutants compared to untreated control cells (Fig 6D). Collectively, these results confirm that reducing functional SPP impairs HSV-1 replication.

## Discussion

Herein, we show for the first time that SPP is required for HSV-1 infectivity and that SPP specifically binds to gK. Similar to this study, it was previously shown that small interfering RNA targeted to SPP reduced the production of infectious HCV particles [55]. In this study by using SPP dominant negative constructs and SPP shRNA we have demonstrated that SPP is indeed essential for viral replication. gK has been shown to interact with both HSV-1 gB and UL20 [57], [58]. However, our pull-down involving gK expressing plasmid rules out the possibility that the gK-SPP interaction is strictly dependent upon complexing with other viral proteins. Furthermore, our pull-down of HSV-1 infected RS cells using total HSV-1 antibody which does not recognize gK failed to precipitate SPP and vice versa.

The role of SPP in the context of HSV-1 infection has yet to be elucidated; however HSV-1 gK is a type III transmembrane protein which contains an N terminal signal sequence utilized for its insertion into the membrane [59], [60]. HSV-1 gK has also been shown to traffic through both the trans-Golgi network (TGN) [52] and the rough endoplasmic reticulum (RER) [46]. To date, all identified SPP substrates are signal peptides which span the ER in a type II topology [2], [61]. However, to our knowledge there is no study showing that SPP cannot catabolize type III membrane proteins. Type III membranes differ from type II in being multi-pass with the targeting signal sequence on the amino terminus. The location of the signal sequence within gK is essential as both deletion of the N terminus and N terminal cleavage via protease reduce HSV-1 virion entry [62]. Recent work suggests that despite SPP having a catalytic preference for Type II membrane proteins, SPP is able to bind to many types of preproteins, signal peptides and misfolded proteins [63]. It is in this context that SPP is associated with quality control in the ER associated degradation (ERAD) pathway [17], [64]. SPP is thought to function as a membrane protease liberating burdensome protein fragments from the membrane [65], [66]. The fate of these released peptides can be degradation, however their role as signaling molecules is emerging [67], [68]. Under these circumstances the possibility exists that SPP also serves to liberate bioactive fragments of viral proteins including those capable of inducing gene expression. This scenario could explain the negative effects on viral gene expression we observed when SPP was reduced via shRNA.

It has also been shown that over-expression of gK in gK-transformed cells collapses the Golgi apparatus into the ER thus inhibiting virion egress, glycoprotein transport, and virus-induced cell fusion [69]. Similarly in this study we also observed physiological signs of ER stress, such as ER aggregation, in cell lines over-expressing gK. The possibility remains that the increase in glycoprotein processing within the cell during the infectious period damages the ER adding to the immunopathology caused by the virus. The implications of ER stress are well documented in human diseases such as diabetes mellitus atherosclerosis, hypoxia, neoplasia and neurodegeneration [70], [71]. In addition, ER stress has been demonstrated as causative in genetic and environmental models of retinal degeneration [72]. Cells have evolved highly conserved mechanisms to deal with ER stress through the unfolded protein response (UPR) whereby functional protein processing is restored or apoptosis is induced [71], [73]. In fact, HSV-1 has counter-evolved processes to sense ER stress and downregulate the UPR to maintain ER homeostasis and prevent apoptosis [74], [75].

In line with the ER stress and the gK-induced collapse of the Golgi apparatus [69], we have previously shown that a recombinant HSV-1 expressing two additional copies of gK induced severe corneal scarring and dermatitis in different strains of mice [49]. Furthermore, we previously demonstrated that immunization of mice with gK, but not with any of the other known HSV-1 glycoproteins, resulted in exacerbation of CS and herpetic dermatitis following ocular HSV-1 infection [29], [30]. As our results clearly demonstrate the SPP and gK can bind and colocalize with one another the possibility remains that the gK interaction with SPP may be involved in the pathology of HSV-1 induced eye disease. Consequently, this gK-SPP interaction may be considered as a specific therapeutic target for the prevention of corneal infection in patients at risk and a reduction in the severity of the CS in patients who have established infections thereby providing an effective treatment for those suffering from the devastating effects of HSK.

## Conclusion

Glycoprotein K (gK) is a hydrophobic protein and is highly conserved between HSV-1 and HSV-2. Studies using insertion/deletion mutants have shown the importance of the gK in virion morphogenesis and egress. We demonstrated previously that immunization of mice with gK, but not with any of the other HSV-1 glycoproteins, resulted in exacerbation of eye disease and herpetic dermatitis following ocular HSV-1 infection independent of mice or virus strain. We also have demonstrated that a recombinant HSV-1 expressing two extra copies of gK exacerbated eye disease in both mice and rabbit, suggesting that gK over-expression is pathogenic. In this study we have shown for the first time that: (1) HSV-1 gK binds to signal peptide peptidase (SPP); and (2) ShRNA against SPP and SPP dominant negative mutants reduced HSV-1 titers *in vitro.* Thus, blocking the interaction of gK with SPP using SPP shRNA should be considered as a potential alternative therapy in not only HSV-1, but other conditions whereby SPP processing is integral to pathogenesis.

## Supporting Information

Figure S1 **Results from bacerial-2-hybrid indicate SPP interacts with gK.** A) BLAST results from a representative clone indicate strong consensus with all four isoforms of SPP. B) Representative sequence alignment of an isolated clone and SPP isoform 1.
(PDF)

Figure S2 **c-myc-gK construct used for gK-SPP binding.** The structure of the wild-type gK molecule of 338 aa is shown with an in-frame insertion of c-myc sequence on C terminus. Positions of N-glycosylation sites are indicated at AA residues 48 and 58. gK construct was inserted into the BamHI site of plasmid pcDNA3.1.
(PDF)

Figure S3 **HA-SPP constructs used for gK-SPP binding and dominant negative transfection.** The structure of the wt SPP molecule of 43.5 kDa is shown with an in-frame insertion of HA sequence and ER retention signal. Asp219 (D219A) and Asp265 (D265A) are SPP dominant negative mutants in which Asparagine (D) at aa positions 219 or 265 was mutated to Alanine (A) and inserted into plasmid pcDNA3.1.
(PDF)

Figure S4 **SPP knockdown by shRNA construct in different cell lines.** Vero, HeLa and RS cells were grown to confluency and transfected with either SPP shRNA or scramble shRNA. After 24 hr, RNA was isolated from each cell line and qRT-PCR was performed as described in Materials and Methods. SPP expression in each cell line was normalized to the scramble SPP shRNA transfected control cells. Each point represents the mean ± SEM from 3 independent experiments.
(PDF)

Figure S5 **Cell vitality in presence of SPP shRNA.** RS cells were transfected with SPP shRNA followed by infection with 0.1 PFU/cell of HSV-1 strain McKrae. Controls were uninfected cells and cells infected with HSV-1 without SPP shRNA. Cells were harvested 24 hr PI, stained with anti-Annexin-V antibody, and FACS analyses was performed as described in Materials and Methods. Shown is a graphical representation of the % of cells undergoing apoptosis in each group.
(PDF)
