# Supplementary material for: Binding of HSV-1 Glycoprotein K (gK) to Signal Peptide Peptidase (SPP) Is Required for Virus Infectivity
Source: PLoS One. 2014 Jan 20;9(1):e85360. doi: 10.1371/journal.pone.0085360 (PMC3896391; doi:10.1371/journal.pone.0085360)
Supplement: Figure S1 — Results from bacerial-2-hybrid indicate SPP interacts with gK. A) BLAST results from a representative clone indicate strong consensus with all four isoforms of SPP. B) Representative sequence alignment of an isolated clone and SPP isoform 1. (PDF) [file pone.0085360.s001.pdf]

A)

NM_010376.4	Mus musculus histocompatibility 13 (H13), transcript variant 2, mRNA
NM_001159551.1	Mus musculus histocompatibility 13 (H13), transcript variant 1, mRNA
NM_001159552.1	Mus musculus histocompatibility 13 (H13), transcript variant 3, mRNA
NM_001159553.1	Mus musculus histocompatibility 13 (H13), transcript variant 4, mRNA

B)

```
Score = 1138 bits (616), Identities = 774/897 (86%), Gaps = 10/897 (1%)

Query  62   TCTCCGTCTACGATTCGTGCTGCGGCGGCCGGANCTGGAGTCGGAGCCCGAGCGCAGNCT  121
            |||||||||||| |||||||||||||||||||| ||||||||||||||||||||||| ||
Sbjct  59   TCTCCGTCTACG-TTCGTGCTGCGGCGGCCGGAGCTGGAGTCGGAGCCCGAGCGCAGCCT  117

Query  122  NGACATGGATTCGGNTGTCAGNGATCCGAANAANGGNAGNGCCGANGCTGGCACNCCAGN  181
             | ||||||||||| |||||| |||||| | || || || ||||| |||||||| ||||
Sbjct  118  CGCCATGGATTCGGCTGTCAGCGATCCGCACAACGGCAGCGCCGAGGCTGGCACCCCAGC  177

Query  182  CAACGGNANNANGCGGGCGNCCTCCACGCCCGAGGGCATCGGGCTGGCCTANGGCAGCNT  241
            |||||| |  | |||| || ||||||||||||||||||||| ||||||||| |||||| |
Sbjct  178  CAACGGCACGACGCGGCCGCCCTCCACGCCCGAGGGCATCGCGCTGGCCTACGGCAGCCT  237

Query  242  NCNGNTCATGGCGCTGNTGNNCATCTTCTTCGGCGCCCTGNGCTCGGTGCGCTGCGCCCG  301
             | | ||||||||||| ||  ||||||||||||||||||| |||||||||||||||||||
Sbjct  238  CCTGCTCATGGCGCTGCTGCCCATCTTCTTCGGCGCCCTGCGCTCGGTGCGCTGCGCCCG  297

Query  302  CGGTNAAGAGCTCTTCGGACATGCCANAAACCATCANCAGTCGAGATGCCGCCCGCTTCC  361
            |||  ||||||||||||||||||||| ||||||||| |||||||||||||||||||||||
Sbjct  298  CGG-CAAGAGCTCTTCGGACATGCCAGAAACCATCACCAGTCGAGATGCCGCCCGCTTCC  356

Query  362  NNATCATCGCCANNTGNANACTCCTGGGGCTCTANCTCTTTTTNAAAATATTCTCCCNGG  421
              ||||||||||  || | ||||||||||||||| |||||||| ||||||||||||| ||
Sbjct  357  CCATCATCGCCAGCTGCACACTCCTGGGGCTCTACCTCTTTTTCAAAATATTCTCCCAGG  416

Query  422  AGTACATCAACCTCTTGCTGTCCATGTATNTCTTCGTGCTGNGGATCCTGNCCNTGTNAN  481
            ||||||||||||||||||||||||||||| ||||||||||| |||||||| || ||| | 
Sbjct  417  AGTACATCAACCTCTTGCTGTCCATGTATTTCTTCGTGCTGGGGATCCTGGCCCTGTCAC  476

Query  482  ANACCATCAGTCNNTTCATNAATAAGTNTTTTCNANNCAANTTCCCAAANCGCCAGNATN  541
            | |||||||| |  ||||| ||||||| ||||| |  ||| |||||||| |||||| || 
Sbjct  477  ACACCATCAGCCCCTTCATGAATAAGTTTTTTCCAGCCAACTTCCCAAACCGCCAGTATC  536

Query  542  AACTGCTCTTCANACAGGGTNTCTGGGGAAAANAAAGAANAGATCATCAACTATGAGTTT  601
            | |||||||||| ||||||  ||||||||||| |||||| ||||||||||||||||||||
Sbjct  537  AGCTGCTCTTCACACAGGG-CTCTGGGGAAAACAAAGAAGAGATCATCAACTATGAGTTT  595

Query  602  GACACTAAGGACCTGGTGTGNCTGNGNCTAAACAANGTNANTGGTGTCTGGTANNTNNTG  661
            |||||||||||||||||||| ||| | |||| ||  ||   ||||||||||||  |  ||
Sbjct  596  GACACTAAGGACCTGGTGTGCCTGGGCCTAAGCAGCGTCGTTGGTGTCTGGTACCTTCTG  655

Query  662  AAGAAACACTGNATTGCNAACAANNTGTCTGGNCTGGNNTTCTCCCTTAATGGGGTAGAG  721
            | ||| ||||| ||||| |||||  ||| ||| ||||  |||||||||||||||||||||
Sbjct  656  AGGAAGCACTGGATTGCCAACAACCTGTTTGGCCTGGCCTTCTCCCTTAATGGGGTAGAG  715

Query  722  CTCCTGCANCTGAACNACGTGAGCACTGGNTGTATNCTGCTCGGAGGACTCTTNANCTAT  781
            |||||||| |||||| ||||||||||||| ||||| ||||||||||||||||| | ||||
Sbjct  716  CTCCTGCACCTGAACAACGTGAGCACTGGCTGTATCCTGCTCGGAGGACTCTTCATCTAT  775

Query  782  GACATCTTCTGGGGTATTCGGCACCAANGTGATGGTGACANTGGCCAAGTACNTTGAGGC  841
            ||||||||||||| ||||||||||||| |||||||||||| ||||||||| | |||||||
Sbjct  776  GACATCTTCTGGG-TATTCGGCACCAACGTGATGGTGACAGTGGCCAAGTCCTTTGAGGC  834

Query  842  ANCAATAAAATTGGTGTTCCCCATGTATCTGCTGGANAAGGGNCTTGNAAGCATACAACT  901
            | ||||||||||||||||||||  |  ||||||||| ||||| |||| ||||| ||||||
```

Fig. S1
