# Supplementary figures and images for: Binding of HSV-1 Glycoprotein K (gK) to Signal Peptide Peptidase (SPP) Is Required for Virus Infectivity
Source: PLoS One. 2014 Jan 20;9(1):e85360. doi: 10.1371/journal.pone.0085360 (PMC3896391; doi:10.1371/journal.pone.0085360)

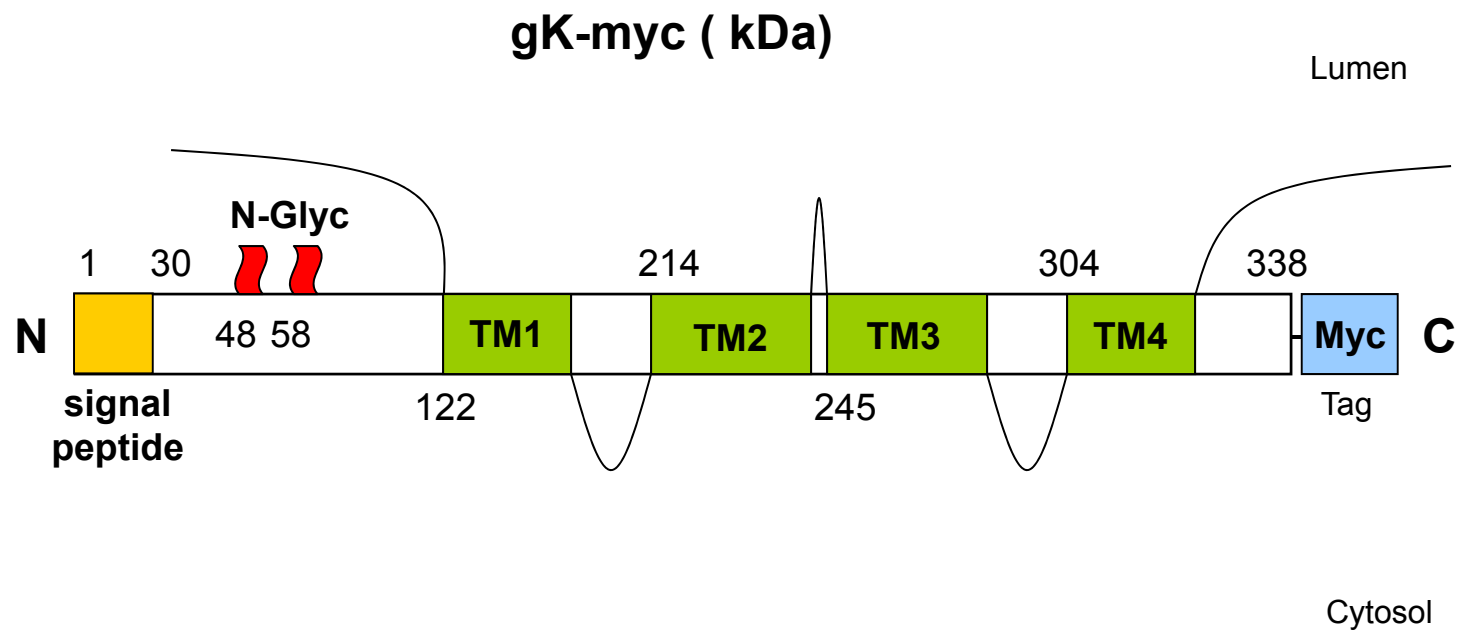

**Fig. S2**

Supplement: Figure S2 — c-myc-gK construct used for gK-SPP binding. The structure of the wild-type gK molecule of 338 aa is shown with an in-frame insertion of c-myc sequence on C terminus. Positions of N-glycosylation sites are indicated at AA residues 48 and 58. gK construct was inserted into the BamHI site of plasmid pcDNA3.1. (PDF) [file pone.0085360.s002.pdf]

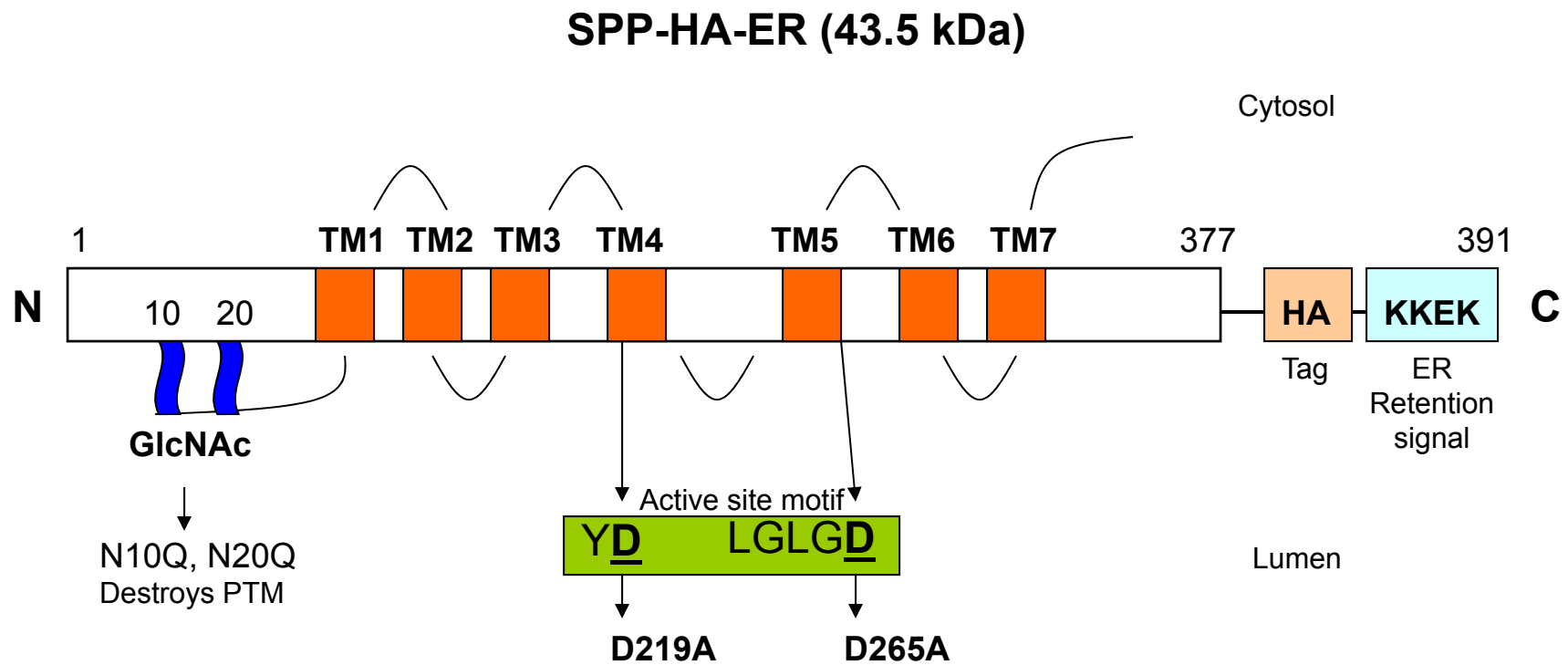

**Fig. S3**

Supplement: Figure S3 — HA-SPP constructs used for gK-SPP binding and dominant negative transfection. The structure of the wt SPP molecule of 43.5 kDa is shown with an in-frame insertion of HA sequence and ER retention signal. Asp219 (D219A) and Asp265 (D265A) are SPP dominant negative mutants in which Asparagine (D) at aa positions 219 or 265 was mutated to Alanine (A) and inserted into plasmid pcDNA3.1. (PDF) [file pone.0085360.s003.pdf]

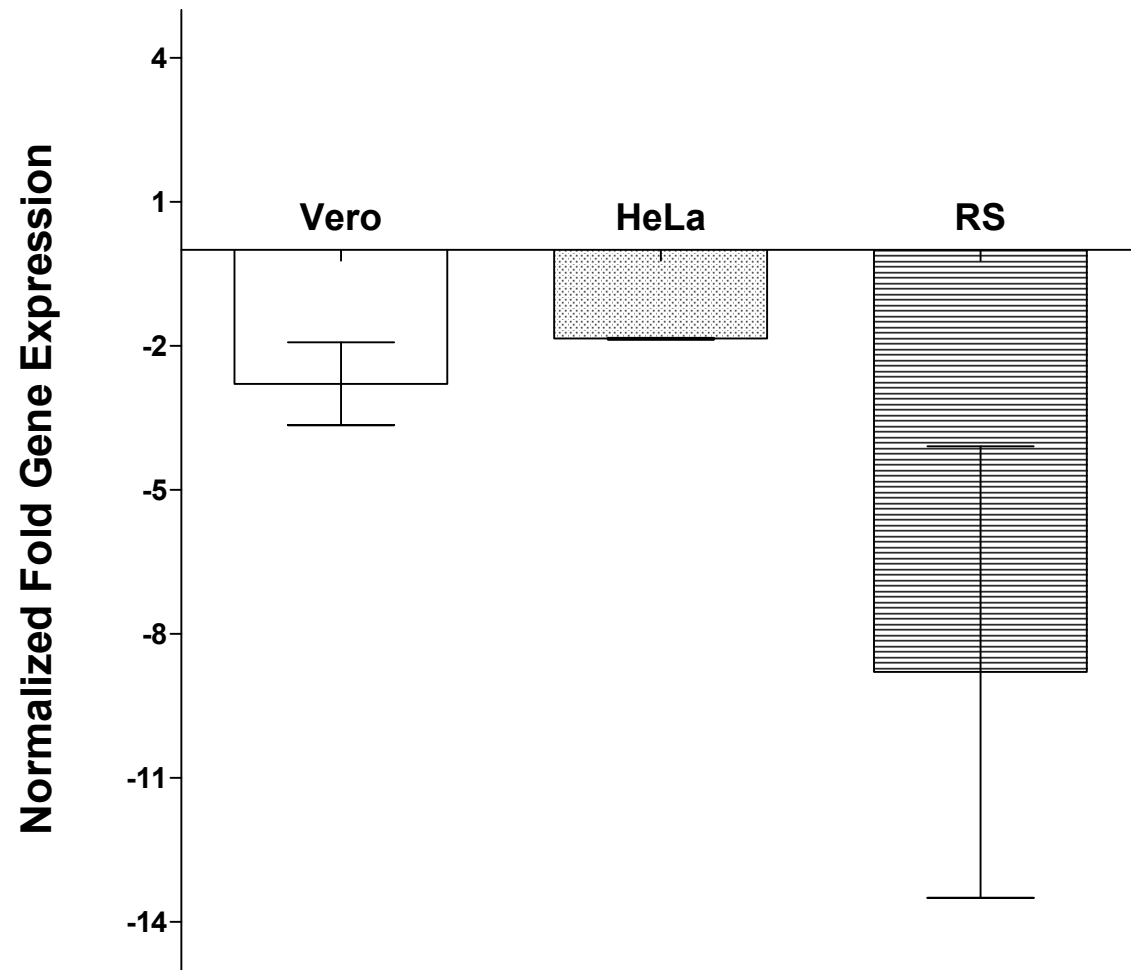

**Fig. S4**

Supplement: Figure S4 — SPP knockdown by shRNA construct in different cell lines. Vero, HeLa and RS cells were grown to confluency and transfected with either SPP shRNA or scramble shRNA. After 24 hr, RNA was isolated from each cell line and qRT-PCR was performed as described in Materials and Methods. SPP expression in each cell line was normalized to the scramble SPP shRNA transfected control cells. Each point represents the mean ± SEM from 3 independent experiments. (PDF) [file pone.0085360.s004.pdf]

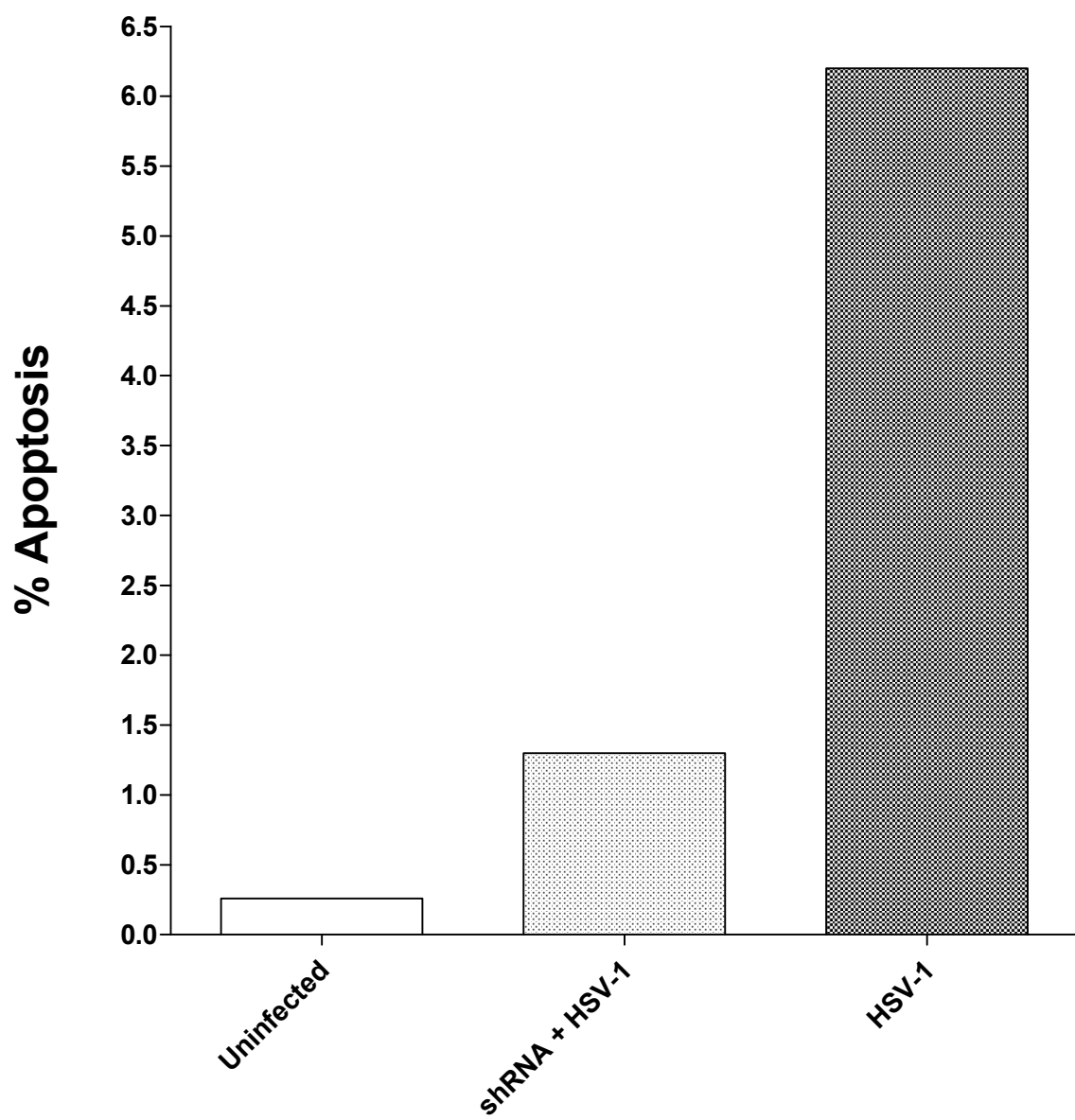

**Fig. S5**

Supplement: Figure S5 — Cell vitality in presence of SPP shRNA. RS cells were transfected with SPP shRNA followed by infection with 0.1 PFU/cell of HSV-1 strain McKrae. Controls were uninfected cells and cells infected with HSV-1 without SPP shRNA. Cells were harvested 24 hr PI, stained with anti-Annexin-V antibody, and FACS analyses was performed as described in Materials and Methods. Shown is a graphical representation of the % of cells undergoing apoptosis in each group. (PDF) [file pone.0085360.s005.pdf]
